# Sustainability capacity and health worker normalisation of a successful non-communicable disease (NCD) health systems intervention within primary care settings in Uganda: a quantitative approach to a qualitative question

**DOI:** 10.1186/s12913-023-09948-w

**Published:** 2023-09-07

**Authors:** David Katende, Norah Nalweyiso, Gertrude Nabulime, Kevin Nakuya, Michael Charles Mubiru, Isaac Sekitoleko, Kathy Baisley, Moffat Nyirenda, Heiner Grosskurth

**Affiliations:** 1 London School of Hygiene and Tropical Medicine, London, UK; 2 MRC/UVRI & LSHTM Uganda Research Unit, Entebbe, Uganda

**Keywords:** Sustainability, Evaluation, Capacity, Chronic diseases, NCDs, Health systems, Primary care, Sub-Saharan Africa, Uganda, Long term, Medium term, PSAT, Normalization, Patient clubs, Adherence clubs

## Abstract

**Background:**

Interventions for non-communicable diseases are increasingly implemented and evaluated in sub-Saharan Africa, but little is known about their medium- to long-term sustainability beyond the end of research funding. A cluster randomised trial conducted between 2013 and 2016 in Uganda and Tanzania showed that an intervention package to improve hypertension (HT) and type-2 diabetes mellitus (DM) care was highly effective in increasing service readiness and quality of care. The present study assesses the sustainability of the intervention 4 years after the trial in Uganda.

**Methods:**

The study was conducted in 2020 in 22 primary care health facilities (HFs) (3 referrals and 19 lower-level units) that had received the intervention package until trial end (2016), to assess their current capacity and practice to sustain ongoing intervention activities for HT and DM care. Through a cross-sectional survey, 4 pre-defined domains *(i.e., cognitive participation, coherence, collective action, and reflexive monitoring)* were examined with regard to health worker (HW) normalization and 8 pre-defined domains for intervention sustainability *(i.e., organisational capacity, local environment, funding stability, partnerships, communication, evaluation, adaptation, and strategic planning)*, using the normalisation tool and the program sustainability tool (PSAT). Summary scores were assessed by domains and facility level.

**Results:**

Overall normalization strength was adequate at 4.0 (IQR: 3.8, 4.2) of a possible 5 with no evidence of association with HF level (p = 0.40); *cognitive participation* (buy-in) and *reflexive monitoring* (appraisal) were strongest at > 4 across all HF levels. All HF levels were weak (< 4) on *collective action* (teamwork) and *coherence* (sense-making). Only collective action differed by level (p < 0.002). Overall intervention sustainability was suboptimal at 3.1 [IQR: 1.9, 4.1] of a possible 7 with weak scores on *funding stability* (2.0), supportive *partnerships* (2.2), and *strategic planning* (2.6). Domain differences by HF level were significant for *environmental support* (p = 0.02) and *capacity in organisation* (p = 0.01). Adequate strength at a cut-off mean of **≥**5 did not differ by HF level for any domain.

**Conclusions:**

Four years after their introduction, practice-dependent intervention elements e.g., local organisational context, HW knowledge or dedication were sustained, but external elements e.g., new funding support or attracting new partners to sustain intervention efforts were not. Whenever new interventions are introduced into an existing health service, their long-term sustainability including the required financial support should be ensured. The quality of services should be upheld by providing routine in-service training with dedicated support supervision.

## Background

Sub-Saharan Africa (SSA) is facing a rapidly increasing burden of non-communicable diseases (NCDs) whilst the prevalence of infectious diseases such as malaria, HIV/AIDS and tuberculosis remains substantial [1, 2]. In Uganda, the prevalence of hypertension (HT) and diabetes mellitus (DM) has been estimated at 26% and 1%, respectively [3] whereas prevalence in the central districts of Wakiso and Mpigi lies between 19 and 26% and 2–4% respectively [4]. The increasing NCD burden has created a demand to incorporate NCD care into health services which in sub-Saharan Africa (SSA) and many other low- and middle-income settings have until recently been structured to mainly manage acute or infectious conditions [5]. Thus, many NCDs go unnoticed and are poorly managed [4, 6].

To address this double challenge, the UN General Assembly issued a resolution on NCDs control and prevention in 2011 stated as “*Resolution 3. Recognize the primary role and responsibility of Governments in responding to the challenge of non-communicable diseases and the essential need for the efforts and engagement of all sectors of society to generate effective responses for the prevention and control of non-communicable diseases;.”* [7] Subsequently, WHO and many governments in low-and-middle-income countries introduced new policies and took initiatives to address the NCD problem including measures to improve NCD care services at primary care level [8]. To facilitate these efforts, several research projects have been launched, including health service-based interventions to improve NCD care, including in SSA [9]. These intervention projects were set at different levels, including hospitals, primary care settings, and in the community, and usually a variety of components including capacity building or task shifting. The effectiveness and fidelity of such research-embedded interventions have been previously reported [9, 10], however little is known about their medium to long term sustainability or effectiveness, e.g., 2 to 5 years after the end of research funding. However, it will be crucial to understand whether newly introduced interventions for chronic NCD care can be sustained in the long term, and how best this can be achieved.

From 2013 to 2016, we conducted a large cluster randomised controlled trial to evaluate the effectiveness of an intervention package that aimed to improve NCD care at primary care facilities in Uganda and Tanzania, with a focus on HT and DM (the health systems and chronic disease project, EACDRP, ISRCTN27340385). The EACDRP trial showed that the intervention was highly effective in improving NCD service readiness at intervention facilities across different levels of primary care, with large and significant differences between intervention and control facilities in the availability of functional basic equipment and consumables and in healthcare worker knowledge. The intervention was also highly effective in improving quality of care, measured by the proportion of NCD patients who were treated according to national guidelines. For example, in Uganda, the mean performance score in intervention facilities was nearly double that in control facilities, and 95% of the intervention facilities provided NCD care according to guidelines compared to only 8% in the control arm [11].

Efforts were also made to ensure that the newly introduced NCD services were sustained after the end of the trial. These included close involvement of the ministerial and local governance structure in study activities throughout the study and handover of important intervention resources (e.g., documents, equipment and up to 9 months of a buffer supply of NCD drugs to overcome potential shortfalls in the national drug supply system). The study also encouraged patient-led initiatives to form patient clubs. These clubs promoted peer support and monetary contributions to a communal fund to procure drugs and other supplies with a high stock out rate, e.g., metformin or glucose test strips. These supplies were issued when the freely provided supplies from the public health system were insufficient.

The EACDRP trial created an excellent platform to assess the medium- to long-term sustainability of a successful health system NCD intervention within primary care settings in Uganda, the **MeLoHanD** study. A comprehensive definition of sustainability of a health intervention includes three components: (i) continued benefits to those who received the health services when the intervention started and extension of benefits to new participants who presented after the supporting funds have been discontinued, (ii) continued implementation of intervention activities by the public health system in which the research had been embedded e.g., a local or national organisation and, (iii) community empowerment to support the continuation of intervention activities after the end of research funding [12].

As part of the MeLoHanD study, we have previously reported the post-trial effects on service availability and readiness and the HF-based quality-of-patient care and experience [13]. We found that supervised aspects of HF performance e.g., the availability of guidelines and records, HW knowledge as well as quality-of-patient care and experience were well sustained. However, logistical aspects of facility performance e.g., the availability of essential drugs and consumables had declined [13].

We also used the MeLoHanD study as an opportunity to assess *HW normalization*, i.e. the degree to which the intervention became incorporated into routine practice [14], and prospective *intervention programme sustainability*, i.e., the degree to which the health system is likely to sustain the intervention efforts in future [15]. This paper presents the results of that assessment of current HW normalization and of the capacity for future intervention sustainability within the MeLoHanD study.

## Methods

### Operational details of the previous trial

The EACDRP trial intervention package included: training of HWs; development of simple clinical guidelines and patient registers; provision of essential NCD care drugs and equipment; active HT/DM case finding among general outpatients (screening); promotion of NCD awareness and screening during community outreaches [11].

At the end of the study in 2016, the HF service readiness and quality of patient care were evaluated [11]. This was done through detailed inspection of each of the intervention and control facilities, including a survey of HWs’ knowledge, and a survey of a random sample of 4 HT and DM patients from each facility. Both assessments used standardised tools and questionnaires [11].

### Study setting

**Fig. 1:**
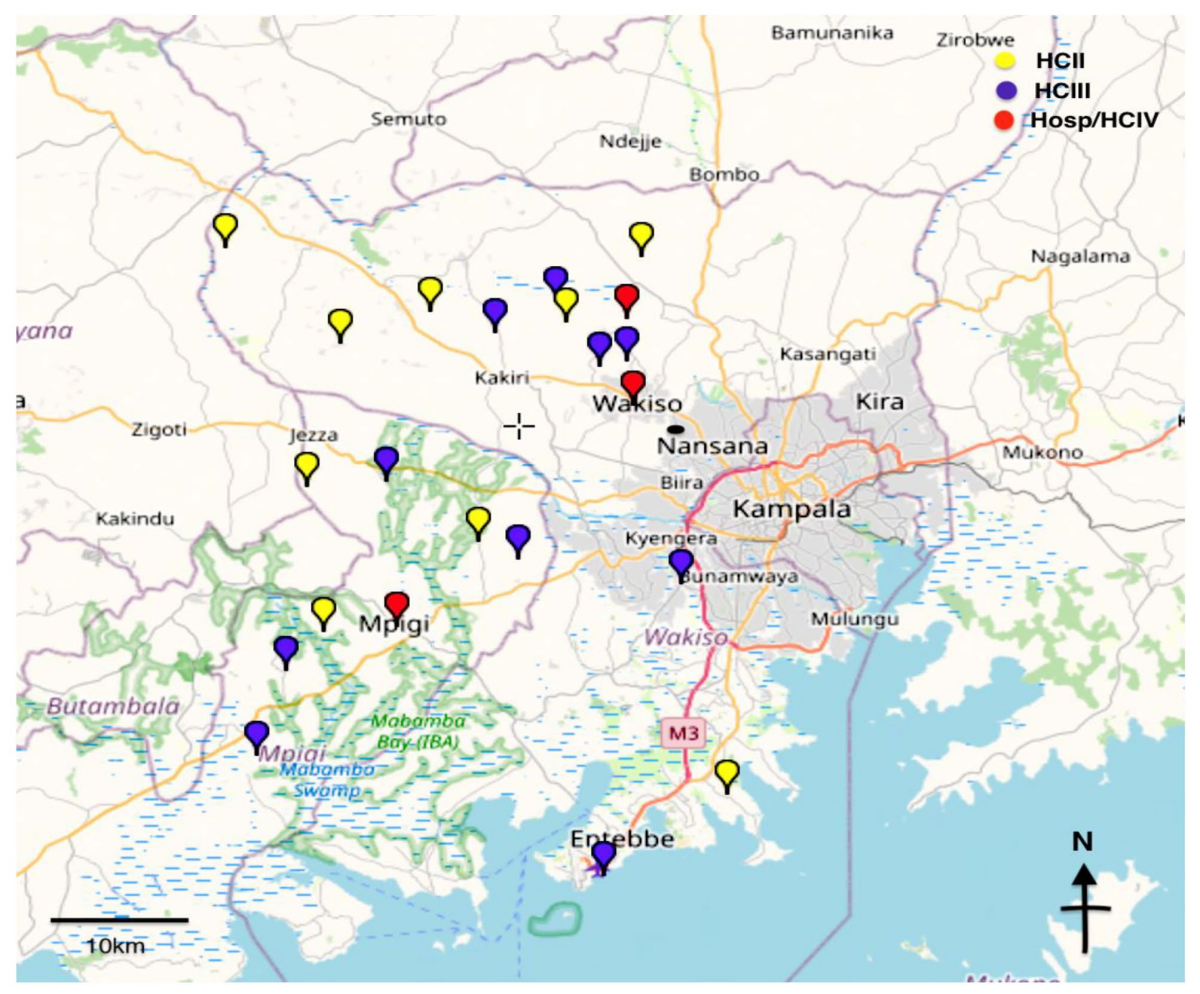
Map showing the distribution of participating health facilities across Mpigi and Wakiso Districts in Uganda (developed using GPS visualizer.com). HC - Health centre levels II, III, IV. Hosp - Hospital

In contrast to the earlier EACDRP trial which had been conducted in Tanzania and Uganda, the current study (MeLoHanD) was conducted between January and December 2020 in Uganda only, in the same two central districts: (a) Wakiso district, which forms a horseshoe shape around the capital city of Kampala and includes urban, peri-urban, and rural areas with a population of 2.5 million; (b) Mpigi district, which lies just southwest of Kampala along the shores of Lake Victoria and has a population of 250,000 (Fig. 1). The population of Mpigi is largely a peri-urban and rural mainly engaged in subsistence farming, fishing, and artisanship.

### Study design

This study involved a cross-sectional survey conducted in 2020 using structured self-administered but supervised interviews of HWs, patients, patient-leaders, and health managers. It evaluated the current degree to which HW *normalization* had been achieved using the normalization tool; and the current capacity for intervention *sustainability* using a validated programme sustainability assessment tool (PSAT) [14–18].

### Description of health facility levels in Uganda

The primary health care system of Uganda is tiered along the politico-administrative organisation of the country (Table 1) and is overseen by the district health office, led by an experienced medical doctor (MD) who co-ordinates resource distribution and staff deployment [19] to health centres II, III, IV and district hospitals (Table 1). Several districts form a region which is served by a regional hospital that can provide specialist care. HCIIs and HCIIIs, which may include some private-not-for-profit health facilities, are expected to diagnose, and manage uncomplicated NCD cases including diabetes, hypertension, asthma, and HIV infection. HCIIs should also be able to diagnose DM, but usually refer DM patients to HCIIIs or higher level facilities [20].

**Table 1:** Description of the Levels of Public Health Service Delivery in Uganda

Health Facility Level^1^	Political or Administrative Level	Catchment Target Population	Main Function or Infrastructural Requirement	Facility head/Supervisor^2^ title and/or their educational background
Regional Hospital	Region or several districts	> 2 million	General and specialist services e.g., ophthalmology	Medical director (e.g., MD + MPH)
District Health Office	District	500,000 to 2 million	Resource distribution, staffing	DHO-er (e.g., MD + MPH)
District Hospital/HCIV	District or constituency	100,000 to 500,000	50–100 in-patient beds, general theatre,	Medical director (e.g., MD+/- MPH)
HCIII	Sub-county	30,000	10–20 in-patient beds, maternity unit, a simple laboratory	Non-MD clinician or Mid-wife
HCII	Parish or several villages	5,000 to 10,000	1–2 day-care beds, first line emergency and out-patient care	Nurse
HCI	Village	1,000–5,000	Mobile outreach post	Nursing assistant or Health visitor

^!^HC- Health Centre; ^2^MD - Medical Doctor; MPH - Master’s course in public health

(Adapted from Readiness of Ugandan health services for the management of outpatients with chronic diseases, Katende et al., 2015)

### Selection of health facilities

This study was conducted in 3 randomly selected higher-level facilities of the originally 6 referral units that participated in the trial, and in all the original 19 lower-level facilities (10 HCIIIs and 9 HCIIs) from the intervention arm of the trial.

There were 7 facilities (4 HCIIIs, 3 HCIIs) in Mpigi and 12 facilities (6 HCIIIs, 6 HCIIs) in Wakiso district. Of these, only Wakiso district (Entebbe) had urban facilities (1 HCIIIs, 1 HCIIs) while the remaining 17 facilities (9 HCIIIs, 8HCIIs) were rural (Table 2).

**Table 2:** Distribution of health facilities by district and facility level

Facility attribute		District				Total
		Mpigi		Wakiso		
		*HCIIIs*	*HCIIs*	*HCIIIs*	*HCIIs*	
Urban HFs	*Singleton*	-	-	1	1	2
Rural HFs	*Singleton*	2	1	3	3	9
	*Paired*	2	2	2	2	8
Referral facilities (HCIVs)		1 *(Peri-urban)*		2 *(1 Peri-urban, 1 rural)*		3
Total		8		14		22

Some HFs had originally been randomised as HCIIIs-HCIIs pairs due to their proximity and to minimise contamination whilst others had been independently randomised (defined here as ‘singleton’). The 2 urban HFs (1HCIIIs, 1HCIIs) were singleton while among the 17 rural HFs − 8 had been selected as pairs (4HCIIIs, 4HCIIs) and 9 (5HCIIIs, 4HCIIs) as singletons (Table 2).

Prior to the start of current study, a pilot study was carried out in two independent HFs (an urban HCIII and a peri urban HCII) to train the study team and to test data collection tools and procedures. Findings from these pilot HFs were used to improve our procedures but were not included in the research dataset of the study itself.

### Selection of participants

All HWs present at the facility on the study visit days were interviewed using the HW normalization tool. On this occasion HWs also took part in the evaluation of service availability and readiness assessment mentioned above and published elsewhere [13].

For the intervention sustainability (PSAT) tool, following experience from the study pilot; only the focal persons at HFs (e.g., HF in-charges, or OPD / NCD clinic heads) rather than all HWs, and likewise only patient leaders (e.g., patient clubs’ leaders or mobilisers) instead of all patients were selected. Health managers at the district and Uganda Ministry of Health (MoH) were also included as well as former intervention officers.

### Data collection and measurement

Interviews were conducted by three trained field workers that had not participated in the previous EACDRP evaluation. They were supervised by an experienced clinician or research nurse.

Data was collected via hand-held tablets using REDCap® version 7.6.3 and actively synced or uploaded on to backup servers at the end of each day. All data entry was overseen by a senior data manager who was also the REDCap programmer.

### Normalization tool

Normalization has been described as the degree to which HWs have managed to routinely embed a new set of activities in already existing knowledge and practices [21]. Normalization can be assessed by applying an instrument (the normalization tool) which was designed to get a better understanding of how to apply and integrate new technologies and complex interventions in health care. The tool asks questions about the implementation of the intervention and is administered to staff with different roles. The tool has 3 parts (A-C) [22]. It has been previously validated [21, 23] and a modified version of this tool and constructs have been used to assess provider-initiated HIV counselling and testing programs in South Africa [24].

Part A – comprises three brief multiple-choice questions about the respondent’s background and their past and current involvement in the intervention.

Part B – comprises three general questions about how familiar the respondent currently feels with regard to the intervention, with a score of 0–10 (maximum score = 30).

Part C – comprises 20 statements regarding the normalization process as perceived by the respondent, with possible responses each ranging from a score of 1 (strongly disagree) to 5 (strongly agree). The 20 statements are ordered under 4 domains, namely:

1. *Coherence (sense making)* – to what extent HWs perceive that the intervention is meaningful to them and their colleagues at the HF (4 statements).
2. *Cognitive participation (buy in)* – to what extent HWs and their colleagues are engaged in the intervention and actively support it (4 statements).
3. *Collective action (active implementation or teamwork)* – to what extent HWs’ individual and team efforts make the intervention work (7 statements).
4. *Reflexive monitoring (appraisal)* – to what extent HWs have access to reports about the intervention and can use this feedback to appraise and improve the intervention (5 statements).

The maximum average score that a HF can achieve under each domain is 5. Across all 4 domains in part C, the maximum aggregate-average score is 20 (4 × 5). Each statement also allows for a lack of response such as a statement not being relevant to their role, not being relevant at the time or not being relevant to the intervention generally.

*More details on this tool can be found at *https://www.rds-se.nihr.ac.uk/wp-content/uploads/NoMAD-questionnaire-for-PPI-with-Logo.docx [22].

### Intervention sustainability tool

Programme sustainability capacity has been defined as *the ability to maintain programming and its benefits over time* [15, 17, 18, 25, 26]. For this work, we used the program sustainability assessment tool (PSAT) to measure this ability. The tool has been validated for use in research and programme settings for chronic diseases [15, 18, 25] and in Africa [26, 27].

This tool assesses the intervention’s current capacity for sustainability across a range of specific organisational and contextual factors. Responses identify sustainability capacity and challenges under three main areas: Programme (Intervention), Organisation and Community [15, 18].

- *Programme (Intervention)* - this refers to the set of formal organised activities that one wants to sustain over time. Such activities could occur at the local, national, or international level and in a variety of settings.
- *Organisation* – this encompasses all the parent organisations or agencies in which the programme is housed. Depending on the programme, the organisation may refer to a national, or local department, a non-profit organisation, a hospital, etc.
- *Community* – this refers to the stakeholders who may benefit from or who may guide the program. This could include residents, organisational leaders, decision-makers, etc.

The tool covers eight domains, and each domain has 5 questions. Responses are scored from 1 (little or no extent) to 7 (to a great extent), giving a maximum score of 35 points per domain and a maximum average score (i.e., from the 5 questions) of 7 points per domain. These domains include:

I. *Environmental support*: having a supportive internal and external climate for the HT and DM intervention e.g., in terms of resources, staffing and drug supplies.
II. *Funding stability*: establishing a consistent financial base for the HT and DM intervention.
III. *Partnerships*: cultivating connections between the HT and DM intervention and its stakeholders, and or interested or affected people or groups.
IV. *Organisational capacity*: having the internal support and resources needed to effectively manage the HT and DM intervention and its activities.
V. *Programme evaluation*: assessing the HT and DM intervention to inform planning and document results.
VI. *Programme adaptation*: taking actions that adapt the HT and DM intervention to ensure its ongoing effectiveness.
VII. *Communications*: strategic communication with stakeholders and the public about the HT and DM intervention.
VIII. *Strategic planning*: using processes that guide the HT and DM intervention’s direction, goals, and strategies.

This questionnaire also allowed for lack of responses e.g., if participants responded that a question was “not applicable” to them or were not able to answer.

More details on the tool can be found at https://sustaintool.org [17] and.

http://creativecommons.org/licenses/by-nc/3.0/.

### Data Analysis

#### Units of analysis

These include HWs, patients, patient-leaders, health managers at HFs, at the district health office and at the MoH as well as former intervention officers. The normalization and sustainability capacity data were not previously collected in 2016, so this analysis was done for the 2020 data only.

#### Sample size

We interviewed all 91 HWs present on the survey dates to determine normalization strength, and 110 individuals (patients, HWs and district/MoH supervisors) to measure intervention sustainability (Table 3). For the sustainability tool (PSAT) and learning from the pilot study, the groups were sub-sampled to only include those directly involved in the day-to-day management of the intervention such as HF managers and/or focal persons (i.e., intervention team leaders at the HFs), and patient leaders (such as patient club leaders or community members of the HF management team) to improve tool precision.

**Table 3:** Distribution of the observed and sub-sampled participants

Groups	Observed overall	Sub-sampled for PSAT
Health workers	91^1^	35^2^
District/ MoH supervisors	11^3^	11^3^
Patients	332	64^2^
Total	**434**	**110**

^1^All 91 HWs that were interviewed for the normalization tool

^2^Only patient leaders or HW focal persons (not all patients or HWs) were interviewed for the intervention sustainability (PSAT)

^3^Includes 3 former intervention supervisors

For the normalisation assessment, assuming a design effect of 2 to allow for the clustering of HW responses within facilities and a standard deviation (SD) of 1.0, a sample of 91 HWs provided > 90% power to demonstrate whether the mean normalization score overall, or for each domain would be > 0.5 higher than 3.5 which is the halfway score rounded up to the next 0.5 (or a hypothesised reference value below the desired target score of ≥ 4 for good domain strength). Adequate domain strength was defined as a score of ≥ 4 of a possible 5.

For the intervention sustainability assessment, with 110 individuals surveyed, and assuming a design effect of 2 and a SD of 1.0, we had > 80% power to demonstrate a similar difference of > 0.4 higher than 4.5 which is the halfway score rounded up to the next 0.5 (or a hypothesized value below the desired target value of ≥ 5 for good domain strength). Hence, adequate domain strength was defined as a score of ≥ 5 of a possible 7.

Within the pre-defined domains, mean and aggregate scores were determined at domain and facility level for both assessments.

The analyses were performed using the statistical package in Stata® version 17.

Graphic or spider-web chart comparisons of domain means or medians by HF level are presented.

Finally, both tools were tested for the internal consistency of all component domains in measuring the outcome using Cronbach’s alpha with a value of ≥0.8 defined as high, 0.6–0.8 as moderate and < 0.6 as low consistency.

## Results

### Assessment of normalization strength

All 91 HWs present on the survey days for the clinical knowledge test in the MeLoHanD study in 2020 [13] were also interviewed for this analysis. This represented 70% of the 131 HWs expected as only 95 HWs were contacted to be met over the 2–3 survey visit days and of which 4 indicated they had transferred out of the HF. Most of the 36 HWs we could not meet, did not attend due to COVID-19 restrictions on HF staffing and travel or absenteeism at the time. Of the 91 HWs, 59 (65%) were female, and 27 (30%) were doctors or clinical officers while 64 (70%) were nursing staff or aides. The median age was 36 (IQR: 31,46). Eighty-two (90%) had been trained during the intervention roll-out from 2014 to 2016 but 23 (25%) of these were not currently involved in NCD case management. Almost all HWs that had not received formal NCD training (8/9) during the original trial reported that they did currently provide NCD care (Table 4).

**Table 4:** Population characteristics of 91 health workers by health facility level and perceptions regarding their engagement in NCD care

Population characteristic	Category		HCIIS (25)	HCIIIS (43)	HCIV (23)	Overall, N = 91	P-value^2^
Age	*Median (IQR)*	37 (33–39)		37 (31–44)	34 (28–40)	36 (31–42)	0.36^3^
Sex	*Women (%)*	18 (72%)		31 (72%)	10 (43%)	59 (65%)	0.10
	*Men (%)*	7 (18%)		12 (18%)	13 (57%)	32 (35%)	
Duration of posting at HF	*3 yrs. or less*	5 (20%)		5 (12%)	3 (13%)	13 (14%)	0.60
	*> 3yrs*	20 (86%)		38 (89%)	20 (87%)	78 (86%)	
HW type	*Medical/Clinical Officer*	0 (%)		13 (30%)	14 (61%)	27 (30%)	**< 0.001**
	*Nurse/Midwife*	14 (56%)		18 (42%)	6 (26%)	38 (42%)	
	*Other*	11 (44%)		12 (30%)	3 (13%)	26 (28%)	
Intervention training and current involvement^1^	*Trained and involved*	20 (80%)		31 (72%)	8 (35%)	59 (65%)	**0.02**
	*involved not trained*	1 (4%)		5 (12%)	2 (9%)	8 (9%)	
	*not involved but trained*	4 (16%)		7 (16%)	12 (52%)	23 (25%)	
	*not involved not trained*	0 (0%)		0 (0%)	1 (4%)	1 (1%)	
Feeling about intervention	*new to somewhat 0–6*	5 (20%)		14 (33%)	8 (35%)	27 (30%)	0.45
	*very familiar = > 7*	20 (80%)		29 (67%)	15 (65%)	64 (70%)	
	*Median scores (IQR)*	10 (8–10)		8 (6–10)	8 (6–10)	9 (6–10)	0.13^3^
“NCD care is now normal part of work”	*not at all to somewhat 0–6*	3 (12%)		10 (23%)	4 (17%)	17 (19%)	0.51
	*completely = > 7*	22 (88%)		33 (77%)	19 (83%)	74 (81%)	
	*Median scores (IQR)*	10 (9–10)		10 (8–10)	9 (7–10)	10 (8–10)	0.24^3^
“NCD care will become a normal part of work”	*not at all to somewhat 0–6*	2 (8%)		6 (14%)	2 (9%)	10 (11%)	0.69
	*completely = > 7*	23 (92%)		37 (86%)	21 (91%)	81 (89%)	
	*Median scores (IQR)*	10 (10–10)		10 (9–10)	10 (9–10)	10 (9–10)	0.45^3^

^1^*‘Involved’* - means they were actively involved in NCD care at the time of this survey

^2^Design-based chi-test of difference between the facility levels

^3^† Kruskal-Wallis’s equality-of-populations rank test

Overall, at HCIIIs and HCIIs there were more female than male HWs, but this was not the case at HCIVs. This gender imbalance was not statistically significant (p = 0.10). However, the type of HW (p < 0.001) and their intervention training attribute (p = 0.02) differed significantly across HF levels. HCIIIs and HCIVs had more clinicians while HCIIs had none. HCIVs had fewer *trained and involved* staff than lower-level HFs (i.e., 35% vs. 71% at HCIIIs and 80% at HCIIs). Interestingly, HCIVs had also the highest proportion of HWs *trained but not involved* (i.e., 52% vs. 16% at HCIIIs or HCIIs). Age, *perceptions about the intervention*, and *whether NCD care was a normal part of their work now or in the future* did not show statistical differences across HF levels.

**Fig. 2:**
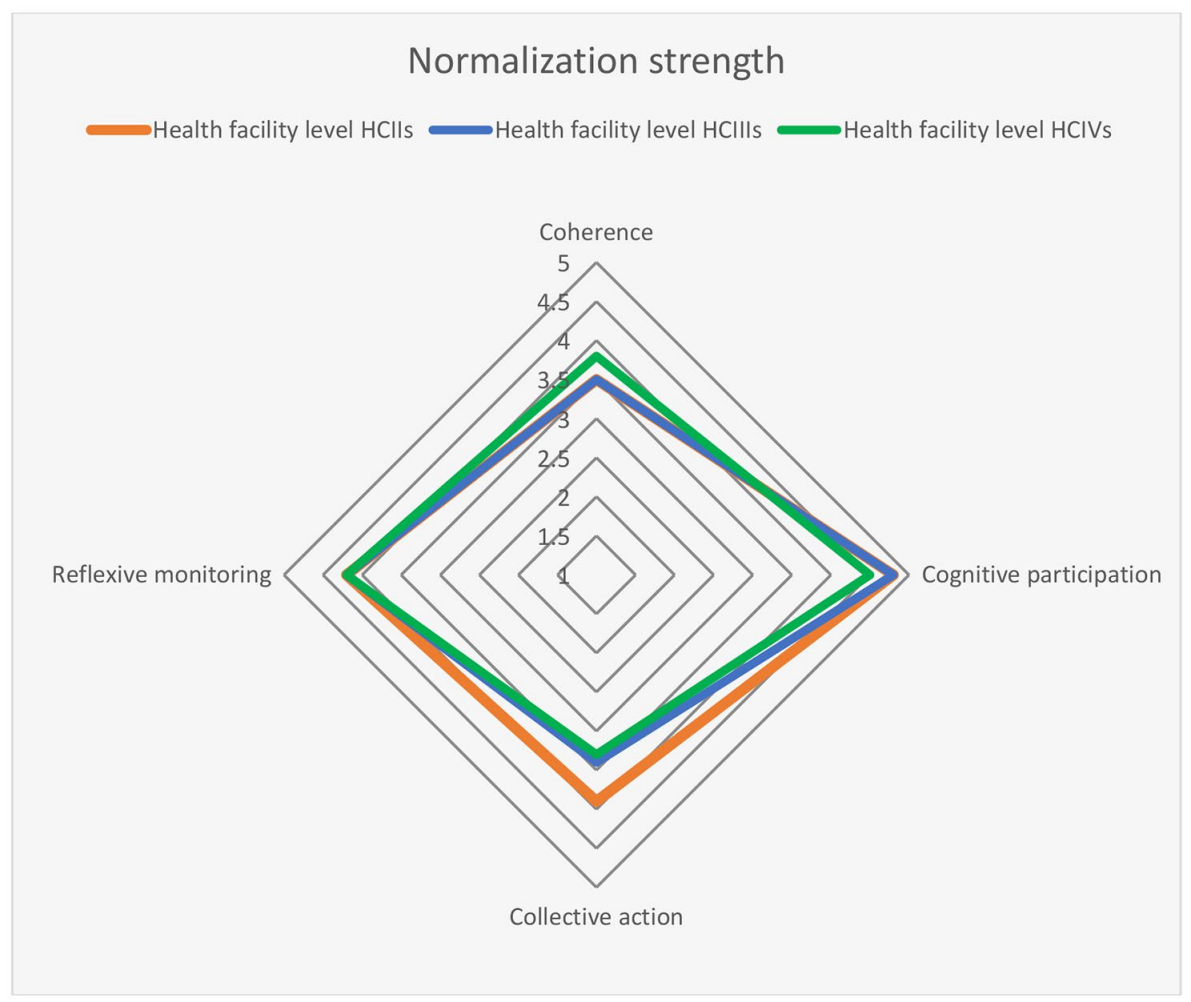
Spider web chart of median scores for the 4 domains of normalization strength, based on responses from 91 respondents to the normalization tool

Overall, the median aggregate score for normalization was 4 out of a possible maximum of 5 (IQR: 3.8, 4.2) with no evidence of association with HF level (p = 0.40). Assessing the four domains with a maximum median score of 5, normalization strength was highest (> 4) for *cognitive participation* and *reflexive monitoring* across all HF levels. With respect to *cognitive participation* more than 95% of all HFs had achieved an adequate level (**≥**4). All HF levels were weak (< 4) on *collective action* and *coherence*; with HCIIs faring strongest on *collective action* at 3.9 (IQR: 3.6, 4.0) while HCIVs were strongest on *coherence* at 3.8 (IQR: 3.5, 4.0) (Fig. 2; Table 5).

**Table 5:** Normalization strength by proportions and medians across domains by health facility level, as reported by 91 HWs, using the normalization tool

Normalization domain	Adequate strength *(Yes≥4/No < 4)* ^*1*^	HCIIs (25)	HCIIIs (43)	HCIVs (23)	Overall domain score, N = 91	p-value^2^
Coherence	*No / NA*	16 (64%)	29 (67%)	15 (65%)	60 (66%)	0.96
	*Yes*	9 (36%)	14 (33%)	8 (35%)	31 (34%)	
	*Median scores (IQR)*	3.5 (3.3, 4.3)	3.5 (3.0, 4.0)	3.8 (3.5, 4.0)	3.5 (3.0, 4.0)	0.22^3^
Cognitive participation	*No / NA*	1 (4%)	1(2%)	0 (0%)	2 (2%)	0.64
	*Yes*	24(96%)	42 (98%)	23 (100%	89 (98%)	
	*Median scores (IQR)*	4.8 (4.3, 5.0)	4.8 (4.3, 5.0)	4.3 (4.5, 5.0)	4.8 (4.3, 5.0)	0.89^3^
Collective action	*No / NA*	14 (56%)	39 (91%)	20 (87%)	73(80%)	**0.002**
	*Yes*	11 (44%)	4 (9%)	3 (13%)	13 (20%)	
	*Median scores (IQR)*	3.9 (3.6, 4.0)	3.4 (3.1, 3.7)	3.3 (3, 3.9)	3.6 (3.1, 4.0)	**0.002** ^3^
Reflexive monitoring	*No / NA*	2 (8%)	8 (19%)	3 (13%)	13 (14%)	0.48
	*Yes*	23 (92%)	35 (81%)	20 (87%)	78 (86%)	
	*Median scores (IQR)*	4.2 (4.0, 4.4)	4.2 (4.0, 4.6)	4.3 (4.0, 4.6)	4.2 (4.0, 4.6)	0.93^3^
Overall facility score, N = 91	*No / NA*	13 (52%)	22 (51%)	11 (48%)	46 (51%)	0.95
	*Yes*	12 (48%)	21 (49%)	12 (52%)	45 (49%)	
	*Median scores (IQR)*	4.0 (3.9, 4.3)	4.0 (3.7, 4.2)	4.0 (3.0, 4.2)	4.0 (3.8, 4.2)	0.40^3^

^1^Assessing domain strength using mean score cut offs Yes≥4 vs. No < 4

^2^Design-based chi-test of difference between the facility levels

^3^† Kruskal-Wallis’s equality-of-populations rank test

IQR – Interquartile range

N/A – “not applicable or relevant” or “not answered”

Only *collective action* was substantially stronger at HCIIs than at HCIIIs and HCIVs (p = 0.002), although still only 44% of HCIIs achieved adequate strength (**≥**4).

### Internal consistency of the normalization tool

In assessing the internal consistency of the normalization tool, each of the four component domains’ mean scores were included as assumed equal maximal contributors to the overall normalization strength (or aggregate mean score). Using a standardised Cronbach’s test of agreement, the tool demonstrated fair internal consistency for normalization strength overall (Cronbach’s α = 0.59) (Table 6). Without reflexive monitoring, the internal consistency of the tool was weakened (Cronbach’s α = 0.37) while without coherence it just slightly improved (Cronbach’s α = 0.62). The other two domains did not appear to affect it much.

**Table 6:** Internal consistency of all four component domains with normalization strength based on responses from 91 HWs using the normalization tool

Domain/Theme	No. of domains^1^	Average inter-domain co-variance (standardised)	Cronbach’s alpha
Coherence	3	0.35	0.62
Cognitive participation	3	0.28	0.54
Collective action	3	0.25	0.51
Reflexive monitoring	3	0.16	0.37
**Normalization strength**	**4**	**0.26**	**0.59**

^1^Assessment excludes the component domain indicated except for the theme “normalisation strength” which includes all 4 domains

### Assessment of intervention sustainability capacity

One hundred ten interviews were analysed. Interviewees included 35 (32%) HW focal persons, 64 (58%) patient leaders and 11 (10%) district/MoH leaders or former intervention officers (Table 3). Thirty-seven (34%) of those interviewed were from urban/peri-urban facilities while 77 (66%) were from rural facilities. Thirty-three (30%) interviews were conducted at HCIIs, 57 (52%) at HCIIIs and 20 (18%) at HCIV or district level or higher.

The overall median domain score was 3.1 [IQR (1.9,4.4)] out of a maximal score of 7; HCIIs showed the lowest overall median capacity at 2.2 [IQR (1.8,3.5)] while HCIVs scored highest at 4.1 [IQR (3.0,4.6)], with HCIIIs just in-between at 3.1 [IQR (1.9,4.3)] (Table 7).

**Table 7:** Median scores for the 8 domains of intervention sustainability, based on responses from 110 respondents to the PSAT

Health facility level	Overall facility median score (IQR)	Sustainability capacity domain (median score (IQR))							
		Environmental support	Funding stability	Partnerships	Organisational capacity	Evaluation	Adaptation	Communication	Strategic planning
HCIIs (33)	**2.2** **(1.8, 3.5)**	2.7 (2.0, 3.8)	1.6 (1.3, 2.6)	2.0 (1.6, 3.2)	2.2 (1.4, 3.7)	2.5 (1.8, 4.8)	2.4 (1.8, 4.4)	2.7 (1.8, 4.7)	1.9 (1.5, 3.6)
HCIIIs (57)	**3.1** **(1.9, 4.3)**	3.4 (2.0, 4.6)	2.1 (1.0, 3.0)	2.2 (1.6, 3.8)	3.3 (1.6, 4.6)	3.4 (1.8, 5.2)	3.5 (2.1, 5.4)	3.9 (2.2, 5.5)	3.0 (1.6, 5.5)
HCIVs/higher (20)	**4.1** **(3.0, 4.6)**	4.4 (3.2, 5)	2.7 (1.8, 3.9)	2.5 (1.7, 4.5)	4.4 (3.7, 5.2)	4.6 (3.5, 5.6)	4.3 (3.1, 5.1)	4.0 (3.1, 5.4)	4.0 (2.2, 5.1)
Overall domain score (110)	**3.1** **(1.9, 4.4)**	**3.2** **(2.0, 4.8)**	**2.0** **(1.3, 3.0)**	**2.2** **(1.6, 3.8)**	**3.3** **(1.7, 4.6)**	**3.4** **(2.0, 5.0)**	**3.2** **(2.0, 5.2)**	**3.6** **(2.2, 5.4)**	**2.6** **(1.6, 5.0)**
p-value^1^	**0.02**	**0.04**	0.05	0.38	**0.01**	0.10	0.19	0.07	0.08

^1^Kruskal-Wallis’s equality-of-populations rank test

HC - Health centre levels II, III, IV

PSAT – Program sustainability tool

**Fig. 3:**
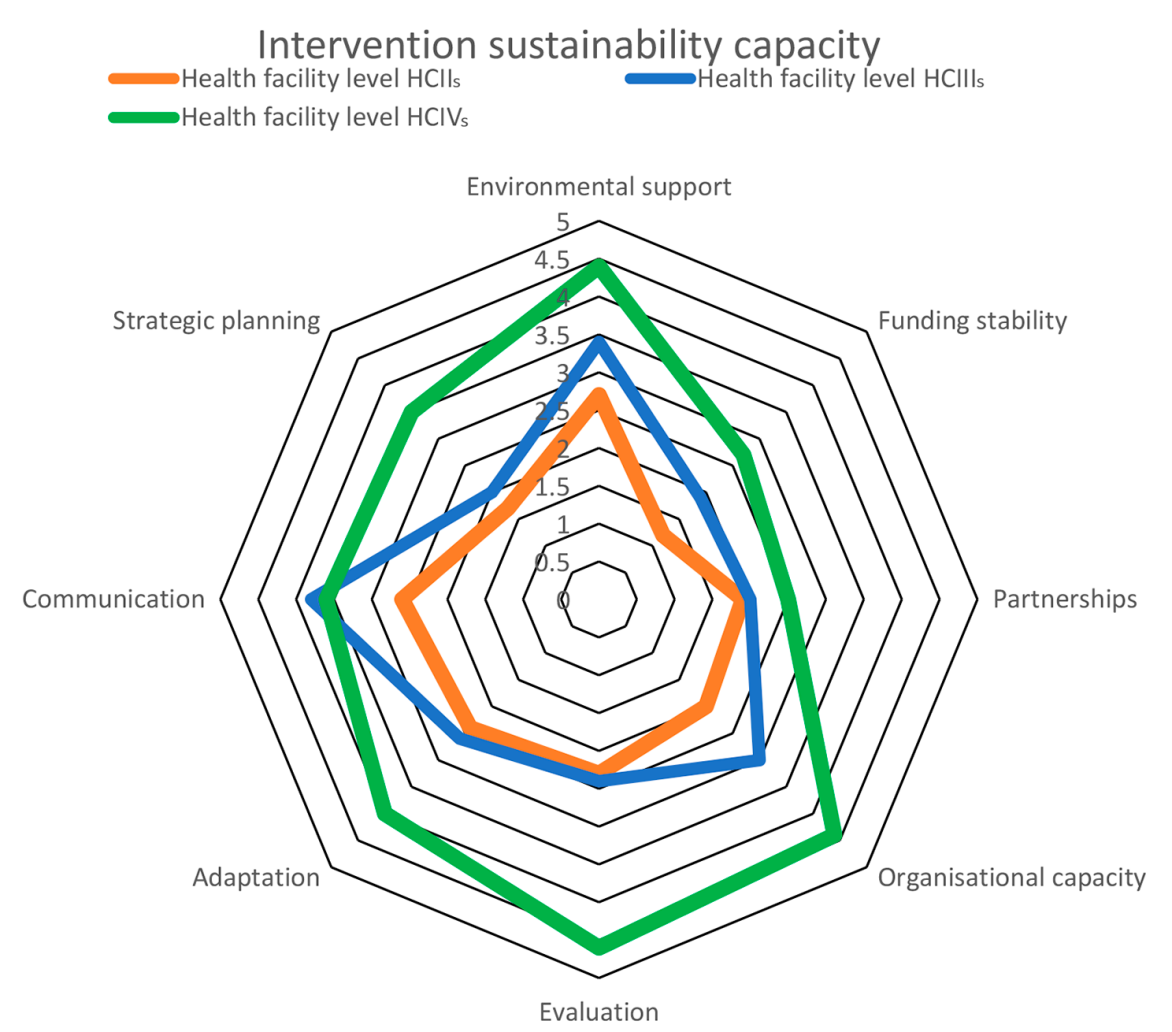
Spider web chart showing median scores for the 8 domains of intervention sustainability capacity, based on responses from 110 respondents to the PSAT

Sustainability capacity was highly dependent on facility level (p = 0.02), with HCIVs scoring higher than HCIIIs which in turn scored higher than HCIIs in nearly all domains. HCIVs demonstrated particularly high scores (> 4) in *environmental support*, *capacity in organisation*, *evaluation*, and *adaptation*, and moderate scores for *communication* and *strategic planning.* HCIIIs were strongest at *communication* (3.9) but of moderate strength (3.1–3.5) at *environmental support*, *capacity in organisation*, *evaluation*, and *adaptation.* HCIIs were mostly weak (< 3) with regard to all domains. The ability to foster *partnerships* and *funding stability* was poor at all facility levels. Evidence for domain differences by facility level was statistically significant for *environmental support* (p = 0.02) and *capacity in organisation* (p = 0.01) and borderline for *funding stability* (p = 0.05), *communication* (p = 0.07) and *strategic planning* (p = 0.08). (Fig. 3)

Using a mean score of 5 as cut off, overall adequate sustainability was highest for *evaluation* (38%) and adaptation (37%), and lowest for *funding stability* (11%) across all HF levels. There was no evidence of a significant difference across facility levels (p = 0.35). *Strategic planning* was proportionately stronger at higher levels (i.e., HCIVs (30%), HCIIIs (44%)) than at HCIIs (21%) while *funding stability* was weakest overall with HCIIs and HCIIIs at 12% each and HCIVs at 5% (Table 8).

**Table 8:** Intervention sustainability by domain and health facility level, based on responses from 110 respondents to the PSAT

Health facility level	Adequate strength^1^ Yes /No (≥5)/ (< 5)		Sustainability capacity domain (N = 110)							
		Environmental support	Funding stability	Partnerships	Organisational capacity	Evaluation	Adaptation	Communication	Strategic planning	Overall facility strength
HCIIs (33)	*No / NA*	27 (82%)	29 (88%)	30 (76%)	28 (85%)	23 (70%)	23 (70%)	25 (76%)	26 (79%)	29 (88%)
	*Yes*	6 (18%)	4 (12%)	3 (24%)	5 (15%)	10 (30%)	10 (30%)	8 (24%)	7 (21%)	4 (12%)
HCIIIs (57)	*No / NA*	43 (75%)	50 (88%)	43 (75%)	43 (75%)	33 (58%)	32 (56%)	36 (63%)	32(56%)	49 (86%)
	*Yes*	14 (25%)	7 (12%)	14 (25%)	14 (25%)	24 (42%)	25 (43%)	21 (37%)	25 (44%)	8 (14%)
HCIVs/ higher (20)	*No / NA*	13 (65%)	19 (95%)	16 (80%)	12 (60%)	12 (60%)	14 (70%)	13 (65%)	14 (70%)	17 (85%)
	*Yes*	7 (35%)	1 (5%)	4 (20%)	8 (40%)	8 (40%)	6 (30%)	7 (35%)	6 (30%)	3 (15%)
Overall domain strength (110)	*No / NA*	83 (75%)	98 (89%)	89 (81%)	83 (75%)	68 (62%)	69 (63%)	74 (67%)	72 (65%)	95 (86%)
	*Yes*	27 (25%)	12 (11%)	21 (19%)	27 (25%)	42 (38%)	41 (37%)	36 (33%)	38 (35%)	15 (14%)
p-value^2^		0.35	0.69	0.30	0.19	0.58	0.47	0.49	0.20	0.35

^1^Assessing adequate domain strength using mean score cut-offs Yes≥5 / No < 5

^2^Design-based chi-test of difference between the health facility levels

HC - Health centre levels II, III, IV

PSAT – Program sustainability tool

N/A – “not applicable” or “not answered”

### Internal consistency of the intervention sustainability tool

In assessing the internal consistency of the intervention sustainability tool, each of the eight component domains’ mean scores were included as assumed equal maximal contributors to the overall intervention sustainability capacity (or aggregate mean score). Using Cronbach’s test of agreement, the tool demonstrated very good internal consistency for sustainability (Cronbach’s α = 0.94) (Table 9). All component domains affected the internal consistency of the tool similarly (Table 9).

**Table 9:** – Internal consistency of all 8 component domains with intervention sustainability, based on responses from 110 respondents to the PSAT

Theme	No. of domains^1^	Average inter-domain co-variance (standardised)	Cronbach’s alpha
Environmental support	7	0.68	0.94
Funding stability	7	0.68	0.94
Partnerships	7	0.68	0.94
Organisational capacity	7	0.65	0.93
Evaluation	7	0.65	0.93
Adaptation	7	0.68	0.94
Communication	7	0.66	0.93
Strategic planning	7	0.65	0.93
**Intervention sustainability**	**8**	**0.67**	**0.94**

^1^Assessment excludes the component domain indicated except for the theme “intervention sustainability” which includes all 8 domain

PSAT – Program sustainability tool

## Discussion

With regard to HW normalization, our study showed that there was generally good or adequate normalization strength (a median score of 4 (IQR; 3.9, 4.3) out of a maximum of 5) at all facility levels. This suggests that some aspects of the EACDRP intervention were well sustained, such that they were now embedded within routine practice. Normalisation scores were particularly high with respect to reflexive monitoring and cognitive participation.

All facility levels showed good strength on cognitive participation which suggests that HWs internalised the intervention’s aims and processes. Evidence for reflexive monitoring was also strong suggesting an ability among HWs to consciously adapt their work to the dwindling support after the end of the trial. The domains of coherence (indicating intervention sense-making) and collective action (indicating ability to work as a team) were weak overall, but unexpectedly more so for higher level units which are usually less affected by absenteeism or the lack of mentors [28, 29]. This might be explained by the fact that larger HW staffing levels [30, 31] may allow for a less rigorous duty schedule and less supervisory oversight or even a reduced opportunity to supervise or be directly supervised by an experienced focal person. In contrast, HCIIs showed greater strength in collective action than higher level HFs. The reason for this, is unclear but the observation may reflect that the necessity to act in a united fashion is particularly strong among small teams.

Most of the respondents (74%) had been previously trained or were fully involved in the post-intervention phase, but this was much less the case at HCIVs than at lower-level HFs (HCIIs & HCIIIs-84% vs. HCIVs-44% - Table 4). This may have contributed to the differences observed on collective action and coherence.

HCIIIs and HCIVs had more clinicians while HCIIs had none which is as expected per current MoH staffing norms. Compared to lower-level HFs, HCIVs had fewer staff who had been trained on NCDs during the trial (e.g., 35% vs. 71% – 80% *‘trained and involved staff’* – Table 4). This was also expected because at referral HFs, only staff directly involved in NCD care at the time of the intervention trial had received the training, for logistical reasons. Most health workers, regardless of HF level, reported either being very familiar with this NCD care intervention (70%) and largely felt it was already part of their normal work (81%) or that it would become so soon (89%).

Whilst we observed evidence for normalization that survived the end of the trial by 4 years, it was also obvious that for some domains, normalization strength was low, suggesting that it may have substantially declined since the end of research-related support in 2016.

It is important to note that normalization is not irreversible, and good practices can be de-normalized over time [14]. Normalization domains are not independent of each other but have dynamic relationships with each other and other domains within the normalization framework of an intervention, such as the organisational context, local context or social norms [14]. Furthermore, normalisation may occur during the course of an intervention project onto some but not all newly introduced activities and procedures [9].

A South African study that examined implementation factors around provider-initiated HIV testing and counselling (PITC) after 2 years of embedding using the normalisation process model found that normalization was promoted by strong senior leadership, implementation support, appropriate accountability mechanisms, an intervention design that adapted to needs and practices, positive staff and patient perceptions, and a responsive organisational context [24]. However, challenges were found in operational weaknesses, patient communication gaps and inadequate training [24]. This is not very different from our findings which showed that HW coherence (sense-making) and collective action as the main weakness at all levels while reflexive monitoring (or intervention appraisal) and cognitive participation (HW buy-in) were the strengths. Similarly, another recent South African study on PITC implementation found that the main facilitator was the participation of all healthcare workers although they also faced barriers such as a lack of workspace and under-appreciation [32]. Another study that explored how solar electrification to off-grid rural primary health care facilities in Ghana and Uganda could improve the availability of maternal and child health services using normalization process theory constructs found that implementation with improved outcomes was associated with stakeholder engagement activities to promote internalization (buy-in or sense-making), provision of materials and information to encourage participation, and establishment of relationships to support integration (or teamwork). Barriers to achieving outcomes were also largely operational such as drug stockouts, lack of transportation and poor amenities.

On intervention sustainability, we found that the overall sustainability capacity was low (median 3.1 (IQR 1.9,4.4) out of a maximum of 7). Higher level units performed better than lower-level ones. Their main strengths lay in communication, evaluation, adaptation and to a lesser extent the local environment or organisational capacity. In contrast, lower-level HFs performed rather weakly with respect to funding stability, forging partnerships, and strategic planning. The disparities between facility levels were particularly high for organisational capacity, evaluation, and strategic planning. This is likely due to differences in organisational capacity that affect strategic management [33, 34] and supervisory support at the different facility levels [35]. The decline in measures of sustainability over the years since the end of the EACDRP trial is likely to be a result of many factors, not only the discontinuation of funding support. These processes have been captured by Chambers et al., in a dynamic sustainability framework, that emphasizes an ongoing dynamism from implementation to continuation or institutionalisation, and from efficacy to effectiveness, with ongoing adaptation from learning and problem solving [36]. More importantly, the framework recognises the fact that as an intervention moves from testing to continuation with little support supervision, a ‘program drift’ occurs (i.e., a decrease in yield or benefit due to deviations from the protocols in operationalised manuals as the intervention is delivered in the ‘real world’) and a ‘voltage drop’ becomes inevitable (i.e., an expected decrease in yield from efficacy to effectiveness into real world use) [36].

Obviously, some domains were better sustained than others. This applies in particular to domains that depended less on funding support and more on good organisation and management, or on staff qualities such as knowledge, confidence, and dedication. There was a paucity of findings within the NCD context in sub-Saharan Africa. However, our observations are similar to those from an NCD programme study in Malaysia, which also applied the PSAT [37]. In that study, seven of the eight domains achieved an average score of ≥ 4: again, with the highest mean scores for communications (4.5) and organizational capacity (4.4). The lowest score was documented for funding stability (3.8) [37]. It is also important to note that as one US study found; participants’ reported PSAT scores about perceived sustainability capacity did not directly align with previously reported perceptions about PSAT domain importance or modifiability and so it might be important to identify potential barriers and enablers influencing program (or intervention) sustainability during the planning phase [38]. A Spanish study that implemented a school-based, peer-led, social-marketing intervention that encouraged healthy diet and physical activity, in low socioeconomic adolescents and examined change in PSAT over time at two periods during intervention implementation: end of the first year and end of the second year found that strategic planning (4.43 +/- 1.98) and funding stability (4.38 +/- 1) were considered deficient domains, and at the end of the second year, these domains had improved by 1.67 points (p = 0.043) and 0.59 points (p = 0.159), respectively. The funding stability increase was not significant, and the sustainability capacity final score was 5.93 +/- 1.13 [39]. The sustainability capacity assessment earlier on in the intervention had allowed its improvement and perhaps even in the long term.

It is unclear whether the modest sustainability capacity that our study found will continue to be maintained in the long term. It will be important to identify ways in which the HFs can maintain or newly establish partnerships. Developing solutions to the lack of funding support will also be essential. The creation of patient clubs might be one option. The organisation of patients into an active and functional club directly impacts funding stability because essential drugs or other critical supplies would become available when freely provided supplies are low. Similar organisational or logistical benefits have been demonstrated with patient adherence clubs in HIV chronic care clinics [40, 41]. Additionally, the organisational structure of clinics and the patient management at HFs can benefit from patient leaders or peer supporters [40].

During the EACDRP intervention, deliberate efforts had been made to encourage sustainability through full engagement of MoH and district leadership in the design and revision of the programme, and by organising regular support supervision to HFs. This engagement, even though to varying extents, has largely continued even with other subsequent research projects.

### Strengths

This study is one of few studies examining factors associated with HW normalization and intervention sustainability among NCD services in SSA. We attempted to use a quantitative approach to answer a qualitative question: how well an NCD service intervention programme was sustained according to the perceptions of primary stakeholders such as HWs, health facility and programme managers, patient leaders, and patients. This approach helped us to quantify the various contributory domains as well as to identify areas of strength or weakness that may be amenable to renewed intervention.

This study used standardised and previously validated tools to explore aspects of normalization and intervention sustainability. Overall, validity testing for both the normalization tool and PSAT showed fair (α = 0.6) to very good-to-excellent (α = 0.9) reliability respectively. From a qualitative viewpoint, this means that we can have confidence in the findings as a true reflection of the perceptions of this study population.

### Limitations

This study was a one-time point cross-sectional assessment as a similar assessment was not done in 2016. Due to lack of this temporal comparison, there is reason to wonder about reverse causality – does current sustainability capacity say more about future capacity (post-intervention) or the previous intervention’s residual capacity? However, the post-intervention period lasted about 4 years which should provide adequate time for honing out of any temporary benefits attributable to the previous intervention. Any benefits still present are probably genuinely institutionalised and should continue to do so well into the future.

Response to scalar score-based questions is usually subjective and prone to respondents choosing the middle ground or null (i.e., between the extreme scores) or a regression to the mean. Additionally, respondents may choose what is perceived as socially desirable or acceptable to them. These were both minimised by allowing for a lack of responses (e.g., if participants responded that a question was ‘not applicable’ or that they did not know the answer). Also, most respondents whether HWs or patients had interacted with the intervention for long which minimised the chance of difference between what they observed and what really prevails [42, 43].

Impact of the COVID-19 epidemic: The COVID-19 outbreak in Uganda represented a challenge to our study. The immediate effect was protraction of the study duration as field activities and data collection had to be suspended for about 4 months. Restrictions on travel and work lasted even longer so that fewer than expected HWs could be interviewed. However, due to the longevity of the intervention we believe that the possible effect of this on the variation of HWs’ responses was small as the majority of HWs (78%) had been based at their health facilities for 3 years or longer.

## Conclusions

About 6 years after the introduction of a multi-faceted NCD health service intervention in Uganda, and 4 years after the end of active research-related funding support, we found that the intervention was still normalized among health workers, at least to some extent. This was particularly the case with cognitive participation and reflexive monitoring at small and mid-level primary care facilities. Higher level primary care units need more supervisory support to improve cognitive participation and to foster teamwork (collective action). In particular lower-level primary care units need support enabling them to strengthen the domain of coherence (sense-making) through improving their organisational capacity and long-term strategic planning. All primary care levels will need to strengthen their evaluation and appraisal capacities to maintain optimal reflexive monitoring.

Regarding intervention sustainability, we found that low and mid-level primary care units generally scored sub-optimally (or < 4) on all 8 domains. Higher level primary care units were weak on funding and with respect to supportive partnerships with other stakeholders. Overall, good funding stability, effective partnerships and long-term strategic planning are needed to ensure continuity in services and logistics at all levels.

Future overall sustainability capacity may be enhanced by maintaining and strengthening supervisory support (e.g., in-service support supervision) and organisational capacity, a better communication strategy and adaptation in the absence of adequate or reliable funding. More studies are needed to understand exactly how and when each of these domains come into play in different settings during the life-course of an intervention and its post-implementation period.

## Data Availability

The datasets that support these findings are available from London School of Hygiene and Tropical Medicine (LSHTM) and MRC/UVRI and LSHTM Uganda Research Unit (MUL), but restrictions apply to the availability of these data, which were used under licence for the current study, and so are not publicly available. However, data are available from the authors (David Katende) upon reasonable request and with permission of both LSHTM and MUL. Contact person: Ayoub Kakande Email: Ayoub.Kakande@mrcuganda.org.

## List of abbreviations

CD(s): Chronic Disease(s)
DM: Diabetes Mellitus
EACDRP: EACDRP – East African Chronic Disease Research Project
GCP: Good Clinical Practice
HC: Health Centre (Is, IIs, IIIs, IVs or 1, 2, 3, 4 levels)
HF(s): Health Facilities
HIV: Human Immuno-deficiency Virus
HSP: Human Subject Protection
HT: Hypertension
HW(s): Health Workers / Healthcare Workers
ICF: Information and Consent Form
IQR: Interquartile Range
LSHTM: London School of Hygiene and Tropical Medicine
MeLoHanD: Medium to Long term sustainability to improve management of Hypertension and Diabetes within the primary care setting in Uganda
MoH: Ministry of Health (Uganda)
MRC: Medical Research Council
MUL: MRC/UVRI and LSHTM Uganda Unit (MUL)
NCD(s): Non-communicable Diseases
OPD: Out-patient Department
PSAT: Program Sustainability Assessment Tool
UVRI: Uganda Virus Research Institute
SSA: Sub-Saharan Africa
SD: Standard deviation
WHO: World Health Organization

## Declarations

### Research conduct and compliance

Research on human subjects or material or data in this study was conducted in compliance the Declaration of Helsinki and followed Good Clinical Practice (GCP) guidelines. All study staff were also GCP trained. Patients with uncontrolled NCDs detected during the outcome assessment at health facilities were jointly discussed with the healthcare provider at their respective facility to ensure that they receive adequate care.
